# Enhanced spatial focusing increases feature-based selection in unattended locations

**DOI:** 10.1038/s41598-018-34424-5

**Published:** 2018-10-31

**Authors:** Mandy V. Bartsch, Sarah E. Donohue, Hendrik Strumpf, Mircea A. Schoenfeld, Jens-Max Hopf

**Affiliations:** 1 Leibniz Institute for Neurobiology, 39118 Magdeburg, Germany; 2 Department of Neurology, Otto-von-Guericke University, 39120 Magdeburg, Germany; 3 Kliniken Schmieder Heidelberg, 69117 Heidelberg, Germany; 4 Center for Behavioral Brain Sciences, Otto-von-Guericke University, 39106 Magdeburg, Germany

## Abstract

Attention is a multifaceted phenomenon, which operates on features (e.g., colour or motion) and over space. A fundamental question is whether the attentional selection of features is confined to the spatially-attended location or operates independently across the entire visual field (global feature-based attention, GFBA). Studies providing evidence for GFBA often employ feature probes presented at spatially unattended locations, which elicit enhanced brain responses when they match a currently-attended target feature. However, the validity of this interpretation relies on consistent spatial focusing onto the target. If the probe were to temporarily attract spatial attention, the reported effects could reflect transient spatial selection processes, rather than GFBA. Here, using magnetoencephalographic recordings (MEG) in humans, we manipulate the strength and consistency of spatial focusing to the target by increasing the target discrimination difficulty (Experiment 1), and by demarcating the upcoming target’s location with a placeholder (Experiment 2), to see if GFBA effects are preserved. We observe that motivating stronger spatial focusing to the target did not diminish the effects of GFBA. Instead, aiding spatial pre-focusing with a placeholder enhanced the feature response at unattended locations. Our findings confirm that feature selection effects measured with spatially-unattended probes reflect a true location-independent neural bias.

## Introduction

Visual attention helps us to select the behaviourally-relevant information from the surrounding environment. The selection process can be based on location (spatial attention)^1–3^, or it can operate at the level of features like colour, orientation, or motion (feature-based attention)^4–10^. A yet unsettled question is how those different forms of attention (i.e., spatial and feature-based) interact. That is, feature-based selection could hierarchically depend on prior spatial selection^4,11–14^, or operate entirely independently of location selection^5,15–18^. The main reason for this ongoing debate is that effects of feature-based attention are quite difficult to disentangle from the spatial selection processes. Specifically, as task-relevant features typically define the target, and the target occupies a spatial location, feature-based and space-based selection usually coincide. As such, separating these two forms of attentional selection requires non-trivial experimental manipulations.

A first step to disentangle the effects of feature selection and spatial attention is to control the influence of spatial selection processes by keeping the attended location constant while manipulating feature selection. This can be done by presenting both attended and unattended feature values at the same location (i.e., by showing overlapping, spatially intermingled groups of dots or bars that differ in colour, orientation or motion direction^9,15,18–20^). With such paradigms, it was possible to show that attention can be switched between feature values – like different colours – without changing the attended location. While this proves that feature selection is a process that can be manipulated independently from spatial attention, those studies cannot say whether feature-based attention hierarchically depends on a previous location selection. If this would be the case, feature selection should not be able to operate at unattended locations, i.e., outside the spatial focus of attention (FOA).

A growing number of experiments, however, support the idea that the selection of features is not confined to the attended location. Visual search experiments revealed that feature attention can operate outside the spatial FOA, as it precedes the spatial selection of the target^21,22^. Psychophysical studies observed tilt or motion aftereffects at locations that did not contain a stimulus in the adaptation period^23–25^, and an fMRI study decoded the attended motion direction from a spatially unattended and even unstimulated region of the visual field^26^. All of these findings are in line with the idea that feature-based attention operates at unattended locations and hence independently of spatial attention across the entire visual field – a phenomenon referred to as global feature-based attention (GFBA). However, it is quite challenging to characterize the neural signature(s) of GFBA. A popular way to retrieve neural correlates of feature-based attention outside the spatial FOA is to compare the brain response to feature probes presented at spatially unattended locations as a function of whether or not the probes match the currently-attended feature value e.g.,^5,6,27^. In the following, we will refer to this experimental approach as ‘unattended probe paradigm’, which has found wide application in single-unit recording studies in monkeys^5,6,28^ as well as in human imaging studies including functional magnetic resonance imaging (fMRI), electroencephalography (EEG) and magnetoencephalography (MEG)^4,7,8,11,12,17,27,29–33^.

Importantly, the correct identification of the neural correlates of GFBA, as described above, relies on the assumption that the spatial FOA is consistently directed to the target location and away from the probe. If the spatial FOA would temporarily encompass the “unattended” probe, the reported GFBA modulations could appear during a transient spatial selection of the probe. Although the target and the feature probe are often presented in opposite visual fields^4,5,7,8,11,12,17,27,29–33^, it could be that the subjects transiently shifted their spatial FOA to the probe upon stimulation onset, or that they initially attended with a wide spatial focus that encompassed both the target and the probe. Critically, initial, transient parts of spatial orienting are suggested to be mediated by bottom-up stimulus features and to be not under voluntary control^34,35^. Especially abrupt visual onsets attract spatial attention independent of the task at hand^36^, unless there is a strong pre-focus on the target location^37,38^. That is, the onset of the unattended feature probe could just cause such bottom-up attentional capture leading to a spatial selection of the probe. This raises the question as to whether the brain response to the probe might sometimes be confounded by spatial selection processes. In addition to this potential confound in unattended probe paradigms, there is no unitary pattern in the neural responses to probes as reported by previous EEG/MEG studies, and it appears these responses differ substantially depending on the experimental parameters used. That is, studies that flashed probes during a continuous target presentation reported early visual processing (P1) effects that probably reflect the suppression of distractor colours^27,31^. Experiments using simultaneous target and probe onset, on the other hand, observed N1/N2-like modulations that start at higher-level extrastriate visual cortex areas and most likely reflect an enhanced target colour processing^8,33^. It is likely that those neural correlates do not index the same GFBA process, since the P1-effect seems to rely on the presence of a competing colour in the spatial FOA, whereas modulations in the N1/N2 time range arise without such competition^8^. Crucially, the studies that found complex modulation sequences in the N1/N2 time range used fairly simple target discrimination tasks that did not require strong spatial focusing^7,8,33^, while those reporting P1 effects employed rather difficult tasks^27,31^. Notably, some studies that required high spatial resolution and strong spatial focusing due to small stimuli and brief stimulus presentations found feature selection to be completely absent or at least substantially reduced outside the spatial FOA^4,11,12^. It is therefore possible that the previously reported complex GFBA modulation sequences in the N1/N2 time range^7,8,33^ do not reflect true location-independent feature selection, but at least partially result from a transient spatial selection of the probe due to inconsistent spatial focusing.

Here, we address this issue with two versions of the unattended probe paradigm previously used in Bartsch, *et al*.^8^, that allow us to manipulate the strength and consistency of spatial focusing onto a colour-defined target, and thereby assess the effect of this manipulation on the previously-reported neural indices of GFBA in the N1/N2 time range. One way to improve subjects’ focusing onto the target is to increase the task difficulty. It is well documented that an increased difficulty of target discrimination narrows the spatial focus and reduces the influence of irrelevant peripheral stimuli^39–42^. In Experiment 1, we therefore motivate stronger spatial focusing by increasing the difficulty of target discrimination in the spatial FOA. Another possibility to enhance spatial focusing is to provide subjects with placeholders before stimulus onset. It has been shown that placeholders serve as visual anchors that can effectively shape the distribution of spatial attention across the visual field^43^ and support anticipatory shifts of attention to the location of the upcoming target^44^. In Experiment 2, we therefore demarcate the target location prior to target presentation with a placeholder to aid and expedite spatial focusing onto the target. Bartsch, *et al*.^8^ reported that unattended probes matching the target colour elicited an early (~200 ms) and a late (~280 ms) modulation in the N1/N2 time range in extrastriate visual cortex. We predict that the current experimental manipulations should eliminate at least early parts of this modulation sequence if they reflect an initial spatial selection of the probe at stimulation onset. Alternatively, if the previously reported effects of GFBA arose truly independently of spatial selection processes, they should remain unaffected by the experimental manipulations.

## Methods

### Participants

Nineteen volunteers participated in Experiment 1 (mean age 26.9 years, age range 24–34 years, 10 female, all right-handed) and nineteen participated in Experiment 2 (mean age 27.3 years, 7 female, age range 21–36 years, one left-handed). All subjects had normal or corrected-to-normal visual acuity, gave written informed consent and were paid for participation (6 €/hour with preparation and measurement lasting 2–3 hours). All methods and procedures were conducted according to the research regulations of the Declaration of Helsinki and approved by the ethics board of the Otto-von-Guericke University of Magdeburg.

### Stimuli and Procedure

#### Experiment 1

**Figure 1:**
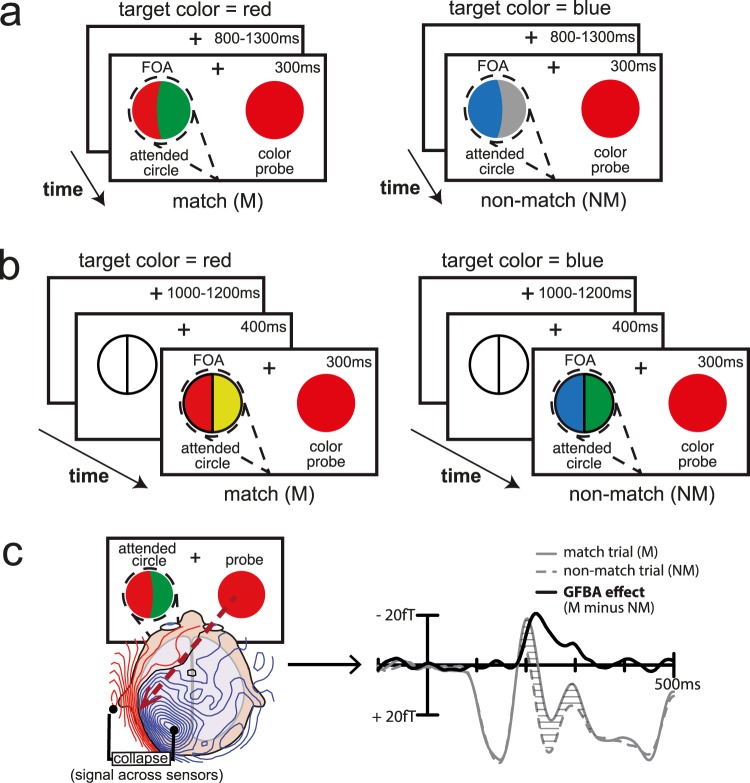
Experimental design. (**a**) Experiment 1. Subjects attended to the bicoloured circle in the left VF (dashed line = spatial focus of attention, FOA) and reported by button press whether the half containing the target colour had a convex or concave shape. The simultaneously presented unattended probe (here: red) in the right VF could either be identical to the target colour (match trial, M) or differ from it (non-match trial, NM). Target colour (red, magenta or blue) was assigned blockwise, probe colour (red, magenta or blue) and distractor colour (yellow, green or grey) changed from trial to trial. (**b**) Experiment 2, placeholder present condition. Subjects attended to the bicoloured circle in the left VF and reported by button press whether its left or right half was drawn in the target colour. Target, probe and distractor colour assignment was as in Experiment 1. To better anchor the subjects’ spatial attention to the location of the upcoming target, the outline of the upcoming bicoloured circle was presented prior to target onset. On half of the trial blocks, the outline presentation was replaced by a blank screen with fixation cross only (placeholder absent condition, not shown here). (**c**) Derivation of GFBA effects. Effects of global feature-based attention (GFBA) were assessed by comparing brain responses to a colour probe (contralateral activity) as a function of whether it currently matched the attended target colour, or not. The M-NM difference served as an index of GFBA. ERMF waveforms show the averaged signal of influx (blue field lines) and efflux (red field lines, polarity inverted prior averaging) magnetic field maxima.

Stimuli: The stimuli of Experiment 1 are illustrated in Fig. 1a. On each trial, two coloured circles were presented, one bicoloured circle in the left visual field (VF) and one unicoloured circle in the right VF. The circles had a diameter of 3.4° visual angle and were placed 4.8° lateral and 2.9° below the central fixation cross. The left circle was composed of two half circles, one drawn in the target colour (red, blue or magenta, assigned blockwise) and one in a distractor colour (yellow, grey or green, randomly chosen trial-by-trial). The vertical midline of the bicoloured circle was slightly bent to the left or right, such that one half of the circle was convex and the other was concave. The position (left or right), as well as the curvature (convex or concave) of the half circle drawn in the target colour (the target), were randomly assigned trial-by-trial. The unicolour circle in the right VF (the colour probe, task-irrelevant) was always drawn in one of the possible target colours (red, blue or magenta, randomly chosen trial-by-trial). The luminance of target, distractor, and probe colours was psychophysically matched prior to the experiment in five independent subjects using heterochromatic flicker photometry^45^, with the average colour luminance being 31 cd/m^2^. All stimuli were presented on a dark grey background (8.3 cd/m^2^) with a white fixation cross (197 cd/m^2^).

Procedure: The subjects’ fixation remained on the central cross while they covertly focused their attention to the target colour half circle in the left VF to report (by button press with the right hand) whether it was convex or concave (convex: index finger, concave: middle finger). The target and the colour probe were simultaneously presented for 300 ms followed by an inter-stimulus interval randomly varying between 800–1300 ms (rectangular distribution). Subjects performed on average 195 (191–198) trials per block and a total of 12 experimental blocks (two easy and two hard blocks for each of the three target colours). Both target colour and difficulty level were designated at the start of each experimental block and alternated such that neither target colour nor difficulty level was repeated on subsequent blocks.

Easy/hard manipulation: The task difficulty was individually adjusted by varying the amount of curvature of the vertical midline in the bicoloured circle and thus, the degree of convexity/concavity of the target half circle. To this end, prior to the main experiment, each subject performed a test block (~8 min) in which they were presented with different levels of convexity/concavity (ranging from −16 to +16°, steps of 2°, each condition shown 20 times). The degrees of convexity/concavity at which the subject performed with approximately 70% and 90% accuracy were used as hard and easy task, respectively. To account for performance changes during the experiment, the amount of curvature was adjusted after each experimental block to respectively maintain these levels of accuracy. On average, the amount of curvature was 19.88° (STD 5.67) for the easy task and 4.23° (STD 2.26) for the hard task.

Trial types: The probe’s colour could either be identical to the target colour (match trials, M) or it could differ from the target colour (non-match trials, NM; see Fig. 1a). Averaged across the different target colours, the subjects performed ~390 match and ~780 non-match trials for each difficulty level (easy/hard). In accordance with our prior work^8^, effects of global feature-based attention (GFBA) were assessed by comparing the brain’s response to the unattended probe circle across the match vs. non-match trials, thereby determining what feature-based attention effects may be present outside the spatial focus of attention. Specifically, non-match trials were subtracted from match trials, which eliminated target-related attention effects (the target was always attended) while it left probe-related attention effects indexing GFBA (probe containing attended color minus probe containing unattended color). Averaging the probe-related attention effects across different probe colours ensured that the observed GFBA modulations were not due to colour-specific effects. See Fig. 1c for a schematic illustration (M-NM difference).

As visible in Fig. 1a, the experimental design enabled us to compare brain responses to physically identical colour probes (same probe colour on match and non-match trials). However, there was still some difference across match and non-match trials, namely that the target colour was present in both visual hemifields on match but not on non-match trials (and was also the most frequently shown colour in a given experimental block). The reason for this is that we (1) decided to keep the target colour constant (and present on every trial) for experimental blocks, such that subjects could build a stable colour bias towards the to-be-attended colour, and that we (2) never used the distractor colour as target or probe (to avoid effects of distractor colour suppression or object-mediated colour attention spreads^46^). Hence, on non-match trials, the probe could never contain one of the colours in the left VF like it was the case on match trials. However, as shown in previous studies, neither the difference in colour balance between match and non-match trials nor low-level effects of colour frequency would give rise to the observed modulation sequence. That is, if subjects had to report the onset of the bicoloured circle independent of its colour, the effects observed with the current paradigm would disappear (see experiment 4 of Bartsch *et al*.^8^). Moreover, even under conditions of a colour attention task, the mere presence of an unattended colour on both sides of the visual field did not elicit GFBA effects (experiment 1 of Boehler *et al*.^46^, separate object condition). All in all, taking attention away from the target colour would eliminate the GFBA effects observed in the current experimental design, which speaks against low-level stimulus properties giving rise to the reported modulation sequence. Another concern could be that the lower number of match trials could have artifically enhanced the amplitude of the match waveform as described by Luck^47^ (pp. 69–71). However, when matching trial numbers between the match and non-match condition (see Supplementary Fig. S3) the same GFBA modulation is observed, which excludes the possibility that M-NM differences were caused by differences in trial number.

#### Experiment 2

Stimuli: The stimuli of Experiment 2 are illustrated in Fig. 1b. Stimulus geometry and colour assignment was identical to that of Experiment 1 except for the following changes: (1) The circles had a diameter of 3.1° visual angle and were placed 4.9° lateral and 3.1° below the central fixation cross. (2) The bicoloured circle in the left VF was drawn with a straight midline and a white outline (197 cd/m^2^). (3) On half of the blocks, the bicoloured circle was preceded by a presentation of its outline serving as a placeholder (placeholder present trials, shown in Fig. 1b). This manipulation led to a low-level physical stimulation difference between trials with and without placeholder in the time range before target presentation. That is, the placeholder will evoke a visual response overlapping with the event-related magnetic field of the following target. While this prevents a direct comparison of trials with and without placeholder, the influence of the placeholder is the same for the match and non-match trials of the placeholder present condition and will thus be eliminated in M-NM difference waveform indexing GFBA (see Fig. 1c).

Analogous to Experiment 1, the luminance of target, distractor, and probe colours was matched prior to the experiment based on heterochromatic flicker photometry^45^ performed by three independent subjects (average colour luminance was 31 cd/m^2^). All stimuli were presented on a dark grey background (8.3 cd/m^2^) with a white fixation cross (112 cd/m^2^).

Procedure: The subjects’ fixation remained on the central cross while they covertly attended to the target colour half circle in the left VF. They were to report via button press with the right hand whether the target colour was located on the left or right side of the bicoloured circle (left: index finger, right: middle finger). On half of the experimental blocks, the outline of the upcoming bicoloured circle was presented 400 ms prior to the target onset thereby providing a visible demarcation of the target location (i.e., a placeholder, shown in Fig. 1b). The placeholder-target interval of 400 ms was chosen according to subjects’ feedback in piloting (shorter durations were not perceived as being helpful and longer as being unnecessarily long). The subjects’ assessment dovetails with evidence from cuing experiments. A psychophysical study shows that the spatial focus of attention needs between 200–500 ms to narrow down onto a target location, and inhibit attentional selection at distant locations (like the probe’s location)^48^. Neural measures of preparatory alpha oscillations suggest as well that subjects need at least 300–400 ms before they build a stable location bias (i.e., alpha lateralization)^49^. Subjects were explicitly encouraged to covertly anchor their attention at the position of the outline prior to target onset to improve their performance. On the other blocks (placeholder absent trials), only the fixation cross was visible during this 400 ms period. For these blocks, subjects were told to attend to the target side, and to still respond as accurately and as quickly as possible. The coloured circles were presented for 300 ms followed by an inter-stimulus interval that randomly varied between 1000–1200 ms (rectangular distribution). The subjects performed 162 trials per block and a total of 12 experimental blocks (two blocks of each target colour (red, blue, magenta) and experimental condition (placeholder absent/present)). The block order was varied between subjects with the constraint that both target colour and placeholder present/absent condition were never repeated on subsequent blocks.

Trial types. The probe’s colour could either be identical with the target colour (match trials, M) or differ from the target colour (non-match trials, NM) (see Fig. 1b). Averaged across the different target colours, the subjects performed 324 match and 648 non-match trials for each trial type (placeholder present/absent). Effects of GFBA were assessed analogously to Experiment 1 (see Fig. 1c).

### Data acquisition

#### Experimental procedure

Subjects were seated in a dimmed, magnetically shielded recording chamber (µ-metal, Vacuumschmelze, Hanau, Germany). Stimuli were back-projected by an LCD projector (DLA-G150CLE, JVC, Yokohama, Japan) onto a partly transparent screen (COVILEX GmbH, Magdeburg, Germany), placed at a viewing distance of 1.0 m. Stimulus presentation was programmed using Presentation (Neurobehavioral Systems Inc., Albany, CA). Subjects gave responses with their right hands via a LUMItouch response system (Photon Control Inc., Burnaby, DC, Canada).

#### MEG recording

Continuous MEG data were recorded with a 248-sensor BTI Magnes 3600 whole-head magnetometer system (4D Neuroimaging, San Diego, CA, USA). The electroencephalogram (EEG) was simultaneously recorded using a 32-electrode cap (Easycap, Herrsching, Germany) with mounted silver/silver chloride electrodes. Respective EEG data are not reported here. Built-in reference coils were used for online cancelation of environmental magnetic noise^50^. To obtain the spatial relationship between the subject’s individual head position and the MEG sensor array, the positions of three anatomical landmarks on the skull (nasion, left and right preauricular points) and five localizer coils (placed on the EEG cap near inion, vertex, nasion, left and right preauricular points) were digitized at the beginning of each experimental session in three-dimensional space using the 3Space Fastrak System (Polhemus, Colchester, VT, USA). Eye movements were tracked by recording the horizontal and vertical electrooculogram (EOG) with electrode placements at the outer canthi of the eyes (bipolar derivation) and an electrode placed below the right eye (unipolar derivation). The analogue MEG and EOG signal was band-pass filtered (DC-100 Hz for Experiment 1 and DC-50 Hz for Experiment 2) and digitized at a sampling frequency of 508.63 Hz for Experiment 1 and 254.31 Hz for Experiment 2.

### Data analysis

#### Epoching and Artifact rejection

MEG data were epoched offline from 200 ms before stimulus onset to 700 ms after stimulus onset. Trials with incorrect, anticipatory (<200 ms) or delayed responses (>1300 ms) were excluded. Artifacts were then rejected by removing epochs in which peak-to-peak amplitude measures exceeded a specific threshold. Thresholds were adjusted individually for each subject in an iterative manner until the data were devoid of major artifacts, leading on average to 9.8% rejected trials with thresholds ranging between 2–4pT (mean: 2.9pT). The remaining epochs were averaged locked to stimulus onset for the different trial types (match and non-match trials) within individual subjects. To increase the signal-to-noise ratio, trial types were averaged across the different probe colours (red, blue, magenta).

#### Repositioning of MEG data

Before computing the grand average data (i.e., averaging experimental conditions across subjects), the data from each subject were repositioned to the most canonical reference head-sensor configuration (selected from 1500 MEG recordings of our lab). Specifically, the subject’s individual leadfield was computed with Curry 7 Neuroimaging Suite (Compumedics Neuroscan, Compumedics USA, Ltd., Charlotte, NC, USA) using the MNI brain (Montreal Neurological Institute brain, ICBM-152 template). The MEG sensor data were then transformed into the source space of the MNI brain by (pseudo-) inverting the leadfield (Miniumum Norm Least Squares approach, MNLS) and afterwards back-projected into sensor-space using the leadfield belonging to the reference head position. As a result, all of the individual MEG data sets were aligned as if they would have been measured with the same reference head position.

#### Current source analysis

For current source localization a distributed source model was estimated from the grand average MEG data using the minimum norm least squares (MNLS) approach as implemented in the Curry 7 Neuroimaging Suite^51^. 3D surface segmentations (boundary element method) of the cerebrospinal fluid space and grey matter of the MNI brain served as the volume conductor and source space compartment, respectively.

#### Event-related magnetic fields (ERMFs)

Determining field effects and choosing sensor sites: To assess the influence of task difficulty (Experiment 1) and placeholder (Experiment 2) on GFBA, sensor sites were chosen to best reflect the GFBA modulation (M-NM difference) independent of other condition-specific effects. Although it is clear that more general effects of easy vs. hard or placeholder present vs. absent on brain activity would be present, these effects are orthogonal to our primary question and thus not extensively examined. In accordance with previous literature, a visual inspection of the data revealed early and late field effects of the match vs. non-match differences in the expected N1/N2 time range^7,8,33^. Since time course and topography were fairly similar between experimental conditions (Experiment 1: easy/hard; Experiment 2: placeholder absent/present), the data were first averaged across those experimental conditions to increase the signal-to-noise ratio and determine time windows and sensors best reflecting the GFBA modulation sequence. To this end, sensors were chosen at the respective early and late field maxima of the overall match minus non-match difference (see Figs 3a and 5a) to retrieve the time course of the underlying GFBA modulations and define time windows of significant match versus non-match differences (see below Statistical validation of amplitude differences). The respective sensors and time windows were then subsequently used to assess GFBA effects for individual conditions, enabling us to compare condition-specific GFBA modulations within the same time range and at the same sensor sites. It should be noted, that choosing sensor sites and time windows on the overall match minus non-match difference waveform (collapsed across easy/hard in Experiment 1 and placeholder absent/present in Experiment 2) is a conservative approach that might miss differences between conditions that would appear when sensor sites were determined based on single conditions. However, as can be seen in the respective result figures, the selected sensors are already at or close to the field maxima of the individual conditions. A re-analysis of the data using sensor sites chosen individually at the maxima of single conditions did not lead to a qualitative change of the reported results (see Supplementary Fig. S2). To account for both efflux and influx components of the event-related magnetic field response, all ERMF waveforms reflect the average of the influx and the polarity inverted efflux response of sensors corresponding with the magnetic field maxima. The grand average ERMF waveforms are plotted from −100 to 500 ms, with the 100 ms period before stimulus onset serving as a baseline. ERMF waveforms were plotted using the Event-related Potential Software Sytem ERPSS (Event-Related Potential Laboratory, University of California San Diego, La Jolla, CA, USA). For visualization purposes only, a smoothing Gaussian filter (low pass, half amplitude cutoff frequency of 23 Hz) was applied. The magnetic fields and headshapes were displayed using the Curry 7 Neuroimaging Suite, headshapes were stylistically modified by M.V.B. for additional clarity.

Statistical validation of amplitude differences: A time-sample by time-sample sliding window t-test on the averaged data of Experiment 1 (easy/hard trials averaged) and Experiment 2 (placeholder absent/present trials averaged) served to find time ranges of significant differences between match and non-match trials. The data were tested with a sliding 30 ms window in the time range of 0 to 500 ms after stimulus onset. To account for the high correlation between time samples in the ERMF data, the number of independent variance components (and not the number of tested time samples) was used to correct for multiple comparisons (Bonferroni correction method). This approach follows the logic of Guthrie and Buchwald^52^, p. 241, which takes the amount of autocorrelation over time as a correction criterion when testing the statistical significance of waveform differences. The number of independent variance components was separately estimated for each experiment. A correlation matrix of all waveforms (time-series of amplitude values) of the reported experimental conditions of all subjects and at all selected sensor sites was subjected to an eigenvalue decomposition. The time samples served as variables. The nominal significance level (here: 0.05) was then divided by the number of eigenvalues >1 (i.e., the number of independent variance components explaining more than one time sample). The tested data of Experiment 1 and 2 contained 16 and 15 independent variance components, respectively, yielding a corrected significance level of 0.0031 for Experiment 1 and 0.0033 for Experiment 2. Pairwise comparisons (t-tests) were then performed on the significant time ranges of early and late GFBA modulations (obtained from the previous step) to test for significant differences between task difficulty levels (Experiment 1) and placeholder absent and present conditions (Experiment 2).

#### Behavioural data

Response time and response accuracy were computed with MATLAB (MathWorks Inc., Natick, MA, USA). Analogous to the MEG data, trials with anticipatory (<200 ms) or delayed responses (>1300 ms) were excluded from further analysis. Response time measures were based only on trials with correct responses. Accuracy and response time effects were statistically validated with the software SPSS (SPSS Inc., Chicago,IL, USA) using repeated measures ANOVAs (rANOVAs). Effects between experimental conditions were further validated using subsequent t-tests. To correct for nonsphericity, Greenhouse-Geisser correction was applied. An alpha of 0.05 served as the significance level.

## Results

### Experiment 1 (easy/hard)

#### Behavioural data

**Figure 2:**
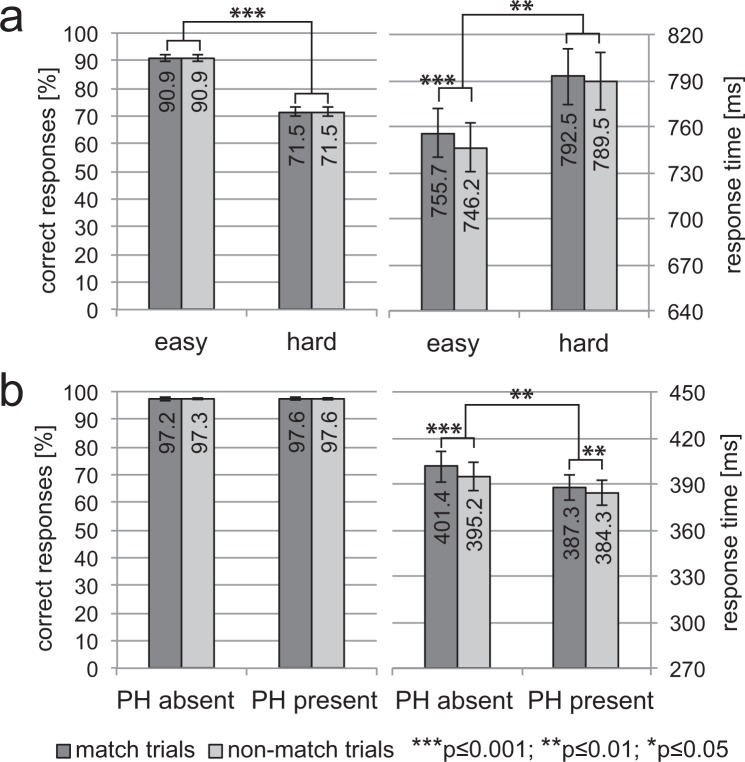
Behavioural Performance. (**a**) Experiment 1. The percentage of correct responses and response times are displayed for both the easy and the hard blocks for match (dark grey) and non-match trials (light grey). Subjects were significantly faster and more accurate when performing the easy task. (**b**) Experiment 2. The percentage of correct responses and response times are displayed for both the placeholder (PH) present and absent condition for match (dark grey) and non-match trials (light grey). While response accuracy was very high with no difference between experimental conditions, the responses were faster in the PH present blocks. In both experiments, responses given on match trials were slightly slower than those given on non-match trials, which is probably caused by an issue of stimulus-response mapping as shown in the Supplementary Data S1. All error bars reflect the standard error of the mean (SEM).

The behavioural performance of Experiment 1 is summarized in Fig. 2a. Response accuracy and response time (RT) are displayed for match and non-match trials of both the easy and the hard task. We conducted two-way rANOVAs with the factors DIFFICULTY (easy/hard) and MATCH (match/non-match). Significant main effects for DIFFICULTY (RT: F[1, 18] = 12.34, p = 0.002, accuracy: F[1, 18] = 167.13, p < 0.0001) revealed that subjects responded significantly faster and more accurately in the easy task (accuracy: 91%, RT: 749 ms) compared to the hard task (accuracy: 72%, RT: 791 ms). For RT, the rANOVAs yielded a significant main effect of MATCH (F[1, 18] = 28.16, p < 0.001) and a significant DIFFICULTY x MATCH interaction (F[1, 18] = 9.88, p = 0.006). Post-hoc pairwise comparisons confirmed that responses were slower on match compared to non-match trials for the easy (p < 0.0001, RT-difference 9 ms) but not the hard task (p = 0.089, RT-difference 3 ms). For accuracy, no significant effect of MATCH (F[1, 18] = 0.001, p = 0.972) or the DIFFICULTY x MATCH interaction (F[1, 18] < 0.001, p = 0.997) were obtained. The slight slowing on match compared to non-match trials refers most likely to an issue at the level of stimulus-response mapping as detailed in the Supplementary Data S1.

### Event-related magnetic field responses (ERMFs)

#### Probe response

**Figure 3:**
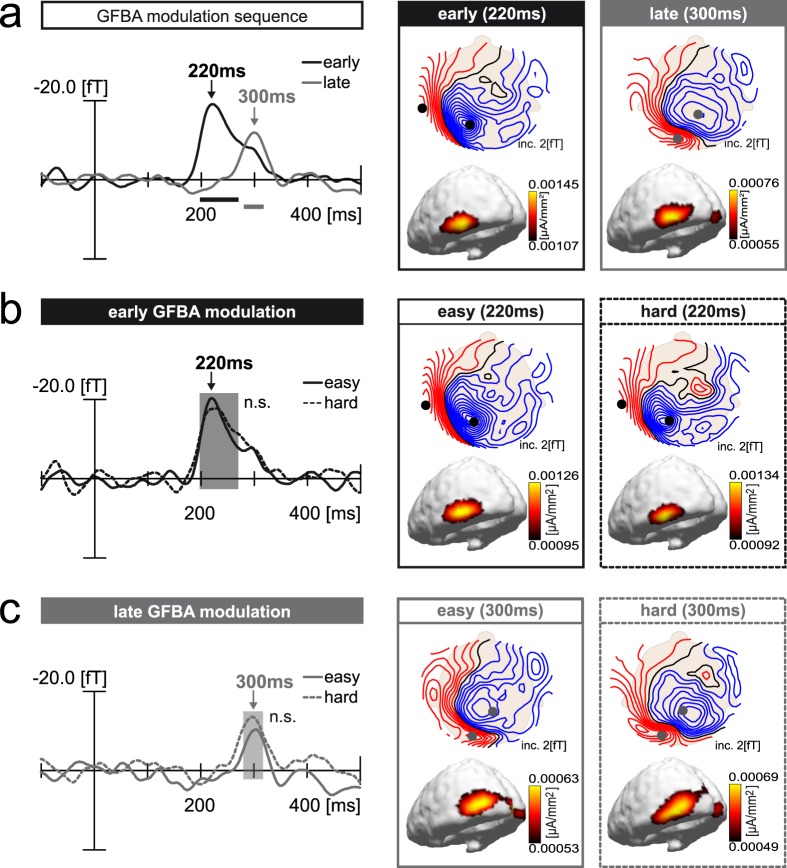
GFBA effects (M-NM difference) of Experiment 1. (**a**) Overall GFBA modulation sequence (match minus non-match difference, averaged across easy/hard conditions). Magnetic field distribution maps (top view, upper row) and corresponding 3D current source density distribution maps (lateral/back view, lower row) at time points of early and late effect maxima are shown on the right. ERMF waveforms of the early (black trace) and late (grey trace) GFBA modulation on the left display the signal of sensors located at the respective field distribution maxima (sensor sites indicated by black and grey dots in the field distribution maps, signal collapsed over influx (blue field lines) and efflux (red field lines, polarity inverted prior averaging) maximum). (**b**) Early GFBA modulation. The waveforms show the early GFBA effect of the easy (black solid) and hard (black dashed) condition. Respective field distribution maps and corresponding current source density distributions are displayed on the right. (**c**) Late GFBA modulation. The waveforms show the late GFBA effect of the easy (grey solid) and hard (grey dashed) condition with corresponding magnetic field maps and current source densities displayed on the right. Sensor sites for the early and late effect (black and grey dots in the field distribution maps) were always chosen at locations of the overall effect maxima (easy/hard average, see (**a**)). Horizontal black and grey bars (**a**) as well as black and grey rectangles (**b**,**c**) indicate time windows of significant match vs. non-match comparisons as determined for the overall GFBA effects (p** < **0.05, corrected for multiple comparisons, as described in Methods).

To derive the overall ERMF modulation indexing GFBA, trials were first averaged across easy and hard conditions before subtracting non-match (NM) from match (M) trials. Figure 3a shows the respective ERMF difference waveforms as well as magnetic field distribution maps and current source density distributions at selected time points after probe onset. The overall GFBA modulation is characterized by an early peak at 220 ms in anterior ventral extrastriate cortex followed by a later peak at 300 ms in more posterior ventral visual cortex. Time ranges of significant modulation effects are highlighted by a black (early peak: 197–270 ms) and grey (late peak: 280–317 ms) horizontal bar. Figure 3b,c compare the early and late GFBA modulation for the easy and hard task. Magnetic field distributions as well as latencies and amplitudes of the ERMF waveforms are fairly similar between the conditions in both the early and late time range. However, while the early GFBA modulation is almost identical, the late modulation seems to be slightly higher in amplitude for the hard task. On time ranges of significant overall GFBA modulations (black and grey horizontal bar, cf. Fig. 3a) t-tests were performed. A pairwise comparison of the two difficulty levels revealed for both time ranges no significant differences (early: p = 0.88; late: p = 0.24). Hence, neither the early nor the late modulation of GFBA was significantly influenced by task difficulty. Additional analyses of the probe response 0–500 ms after stimulus onset also found no interaction between task difficulty and probe match outside the pre-defined GFBA time ranges (see Supplementary Data S2).

#### ERMF response to the target as a function of task difficulty

**Figure 4:**
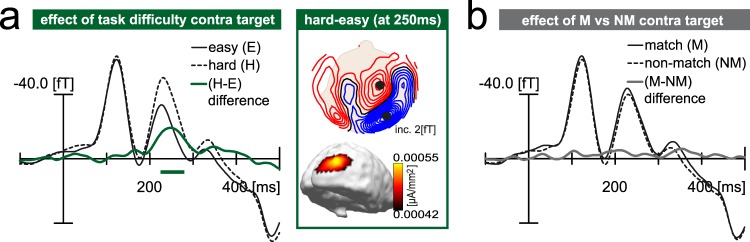
ERMF response to the target (Experiment 1). (**a**) Effect of discrimination difficulty on target-related effects. The waveforms show the brain response contralateral to the target on easy (E, black solid) and hard (H, black dashed) trials as well as the hard-minus-easy difference (green solid). The maps on the right show the field distribution and current source density estimate for the hard-minus-easy difference at the respective modulation maximum. The posterior parietal brain response to the discrimination target is significantly enhanced for the high discrimination difficulty targets (hard task) between 225ms-280ms as indicated by the green bar (p** < **0.05, corrected for multiple comparisons, as described in Methods). (**b**) The waveforms show the brain response contralateral to the target (same sensors as in (**a**)) for match (M, black solid) and non-match (NM, black dashed) trials as well as the match-minus-non-match difference (grey solid). Sliding window t-tests reveal no significant modulation of the target response by a colour match between the target and probe.

When comparing easy and hard trials (averaged across match and non-match conditions), an increased brain response appears contralateral to the target at 200–300 ms after stimulus onset arising in right posterior parietal cortex (see Fig. 4a). The target response did not differ between match and non-match trials (see Fig. 4b). A time-sample by time-sample sliding 30ms-window t-test confirmed a significant difference between easy and hard trials between 225–280 ms (p < 0.05, corrected for multiple comparisons, as described in Methods), but no significant difference between match and non-match trials.

### Summary of Experiment 1

We manipulated discrimination difficulty in the FOA by using a task that required varying levels of fine spatial discrimination, and thus reinforced strict focusing on the target as the discrimination difficulty increased. Any part of the GFBA modulation sequence that is caused by a spatial selection of the probe due to a weak, more diffuse spatial focus should be diminished under conditions of high discrimination difficulty. The behavioural data confirm that the hard task was more challenging for the subjects as indicated by slower and less accurate responses. Furthermore, subjects showed a stronger right posterior parietal brain response to the target when performing the hard task, which is consistent with stronger top-down control signals for focusing attention towards the target^53^ and was independent of whether target and probe colour did match. Nevertheless, the neuromagnetic data do not show a decrease in the brain’s response to the unattended probe. In fact, there is no influence of task difficulty in the early time range of GFBA. In the late time range, the modulation seems to be even larger for the hard task, though this effect was not statistically significant. Taken together, these findings render an explanation of the GFBA effects in terms of allocation of attention to the probe unlikely. However, although the increasing task difficulty motivated subjects to better focus their attention on the target during the convexity/concavity discrimination, the subjects’ spatial FOA might not have been consistently directed to the target location between trials. As a consequence, the onset transient of the probe would have been able to capture attention^37,38^. To address this possibility, Experiment 2 aids spatial focusing by presenting a placeholder immediately prior to the onset of the target and probe.

### Experiment 2 (placeholder present/absent)

#### Behavioural data

The behavioural performance of Experiment 2 is summarized in Fig. 2b. Response accuracy and response time (RT) are reported for match and non-match trials of the placeholder present and placeholder absent condition. Two-way rANOVAs were conducted with the factors PLACEHOLDER (present/absent) and MATCH (match/non-match). A significant main effect of PLACEHOLDER for RT but not accuracy (RT: F[1, 18] = 14.03, p = 0.0015, accuracy: F[1, 18] = 2.58, p = 0.13) revealed that subjects responded equally accurately but significantly faster in the presence of a placeholder (accuracy: 98%, RT: 385 ms) compared to trials without placeholder (accuracy: 97%, RT: 397 ms). For RT, the rANOVA yielded a significant main effect of MATCH (F[1, 18] = 23.18, p = 0.0001), and a significant PLACEHOLDER x MATCH interaction (F[1, 18] = 5.76, p = 0.028). Post-hoc pairwise comparisons confirmed a slight response slowing for match compared to non-match trials that was less pronounced in the placeholder present (p = 0.0069, RT-difference 3 ms) compared to the placeholder absent condition (p = 0.0002, RT-difference 6 ms). As in Experiment 1, the slowing on match trials most likely reflects an issue of stimulus-response mapping (see Supplementary Data S1). For accuracy, the rANOVA obtained no significant main effect of MATCH (F[1, 18] = 0.003, p = 0.96), or the PLACEHOLDER x MATCH interaction (F[1, 18] = 0.02, p = 0.89).

#### Event-related magnetic field responses (ERMFs)

**Figure 5:**
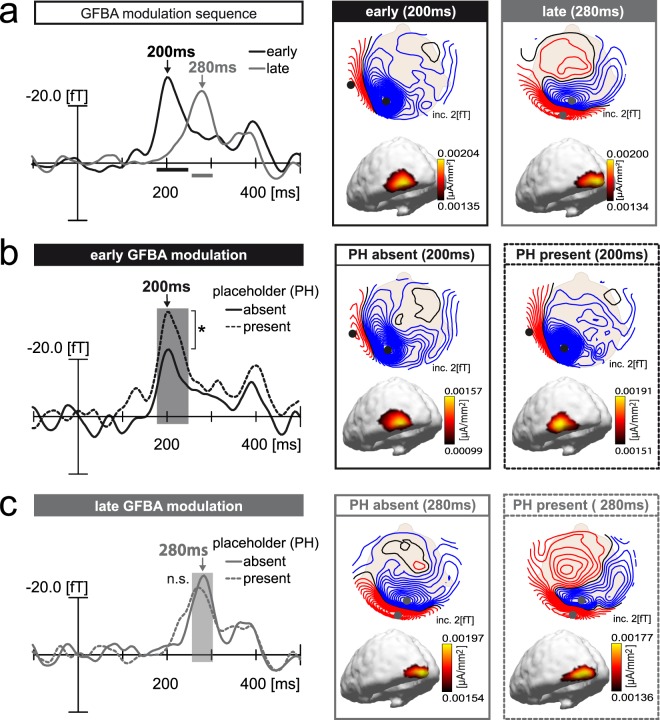
GFBA effects (M-NM difference) of Experiment 2. (**a**) Overall GFBA modulation sequence (match minus non-match difference, averaged across placeholder (PH) absent/present conditions). Magnetic field distribution maps (top view, upper row) and corresponding 3D current source density distribution maps (lateral/back view, lower row) at time points of early and late effect maxima are shown on the right. ERMF waveforms of the early (black trace) and late (grey trace) GFBA modulation on the left display the signal at sensor sites (black and grey dots) chosen at the respective field distribution maxima. Influx (blue field lines) and efflux (red field lines) maxima were averaged after inverting the polarity sign of efflux. (**b**) Early GFBA modulation. The waveforms show the early GFBA effect of the PH absent (black solid) and PH present (black dashed) conditions. Respective field distribution maps and corresponding current source density distributions are displayed on the right. (**c**) Late GFBA modulation. The waveforms show the late GFBA effect of the PH absent (grey solid) and PH present (grey dashed) conditions. Corresponding magnetic field maps and current source densities are displayed on the right. Sensor sites for the early and late effect (black and grey dots in the field distribution maps) were always chosen at locations of the overall effect maxima (PH absent/present average, see (**a**)). Horizontal black and grey bars (**a**) as well as black and grey rectangles (**b**,**c**) indicate time windows of significant match vs. non-match comparisons as determined for the overall GFBA effects (p < 0.05, corrected for multiple comparisons as described in Methods).

Probe response: Analogous to Experiment 1, the placeholder present and absent trials were first combined to derive the overall GFBA modulation sequence (M-NM difference). Figure 5a displays the corresponding waveforms as well as magnetic field maps and current source density distributions. Again, an early modulation maximum (200 ms) in anterior ventral extrastriate cortex is followed by a late, more posterior one (280 ms). Significant time ranges of the match vs. non-match difference are indicated by black (early peak: 178-248 ms) and grey (late peak: 256-304 ms) horizontal bars. Figure 5b,c show the early and late modulation separately for placeholder present and absent trials. While the ERMF time courses and magnetic field distributions are comparable between both experimental conditions, the early ERMF amplitude is apparently higher when the placeholder is present. The late modulation, on the other hand, appears to be slightly weaker compared to the placeholder absent condition. Pairwise comparisons (t-tests) between the placeholder present and absent conditions were computed on the time ranges of significant overall GFBA modulation (black and grey horizontal bar, cf. Fig. 5a). They revealed a significant difference for the early (p = 0.004), but not the late time range (p = 0.47), confirming the stronger early GFBA effect in the presence of a placeholder. Additional 30ms-sliding-window analyses between 0–500 ms confirmed that there was no interaction of placeholder presence and probe match before or after the early GFBA time range (see Supplementary Data S2).

Target response: The placeholder present and the placeholder absent conditions differed in terms of low-level physical stimulation, with the placeholder evoking an ERMF that overlaps with the ERMF response to the target. This is not an issue when comparing match minus non-match difference waveforms (indexing GFBA) of placeholder present and absent conditions since physical differences are the same for match and non-match trials and thus eliminated by the subtraction. A direct comparison of target-related effects between the physically different placeholder present and absent trials, however, would be of limited informative value and is not reported here.

#### Summary of Experiment 2

The presentation of a placeholder (the target’s outline) prior to target onset was designed to enable subjects to better focus and keep their spatial attention on the location of the upcoming target. Subjects made, indeed, significantly faster responses when the placeholder was present, indicating a better (pre-)focusing to the target location. If the early GFBA modulation would arise due to insufficient spatial focusing at the beginning of the trial (i.e., spatial attention being attracted by the probe onset), the early modulation should be diminished in the placeholder present condition. The neuromagnetic data reveal the opposite: the early GFBA modulation increases when the placeholder is present to anchor the spatial FOA to the target’s location before target onset. While the stronger early modulation is followed by a seemingly faster and somewhat weaker late GFBA phase, there was no statistically significant effect of placeholder presence in the late time range.

## Discussion

The reported experiments examined the impact of spatial focusing on ERMF correlates of global feature-based attention (GFBA) measured with the unattended probe paradigm. Our goal was to clarify whether previously reported indices of GFBA in the N1/N2 time range might reflect effects of a transient spatial selection of the probe rather than a global, location-unbound, feature bias outside the spatial FOA. To this end, we manipulated the design of Bartsch, *et al*.^8^ to motivate stronger (Experiment 1) and more consistent (Experiment 2) spatial focusing to a colour-defined target to assess whether the ERMF modulations indexing GFBA would be eliminated. In both experiments, we found robust effects of global feature-based selection. Probes containing the attended colour elicited the previously documented^7,8,33^ modulation sequence in the N1/N2 time range in ventral extrastriate visual cortex. Specifically, we observed an early anterior ventral extrastriate modulation maximum (peaking around 200 ms) followed by a later more posterior ventral extrastriate maximum (peaking around 300 ms). Importantly, motivating a stronger focusing to the target and away from the probe did not significantly reduce any part of the modulation sequence. Moreover, using a placeholder to allow consistent pre-focusing to the target even increased early portions of the GFBA modulation, which is incompatible with the notion that the initial portion of the GFBA modulation arises due to a transient spatial selection of the colour probe. Hence, the results of both experiments provide converging evidence that GFBA modulations measured with the unattended probe paradigm reflect a true spatially global spread of a selection bias for the attended colour.

### Influence of task difficulty on GFBA (Experiment 1)

In Experiment 1, increasing the discrimination difficulty did not significantly alter the GFBA modulations elicited by the unattended colour probe. This makes it unlikely that respective modulations arose because the subjects had a weak focus onto the target, such that the probe occasionally or always appeared in the spatial FOA. In fact, under conditions of high discrimination difficulty, the GFBA modulation showed a slight (non-significant) enhancement in the late time range. That is, if anything, stronger focusing enhanced rather than diminished colour selection outside the spatial FOA. It is reasonable that directing a greater amount of attention to the target in the hard task would entail an enhanced response of neurons involved in the discrimination of the target colour^54–56^. Given that colour selection operates spatially globally, this would explain an increased response also to the probe matching the target colour. Notably, our previous interpretation that late but not early parts of the GFBA modulation are driven by feature discrimination processes^7,8^ reconciles with the slight enhancement for high discrimination difficulty being observed in the late time range.

### Influence of pre-focusing on GFBA (Experiment 2)

In Experiment 1, the subjects might have not been able to consistently anchor their covert spatial focus to the precise location of the upcoming target. Therefore, it is possible that the GFBA effects seen, particularly those that arose as early as 200 ms after target onset, could still reflect some initial spatial selection of the probe. Experiment 2, therefore, provided subjects with a placeholder to pre-focus their attention to the target’s location. Here, the probe still elicited an early and a late modulation, and instead of diminishing it, pre-focusing actually enhanced the early GFBA modulation. Such response enhancement speaks clearly against this modulation reflecting the initial spatial selection of the probe. But why did pre-focusing to the target location enhance the early probe-related response? If feature selection occurs in a spatially global manner, enhanced target selection would – as in Experiment 1 - entail an increase in the feature-related brain responses to the distant probe. Unfortunately, due to physical differences of the visual stimulation on the target side, we cannot directly compare the target responses of the placeholder present and absent condition in Experiment 2. Nevertheless, it is likely that pre-focusing to the target placeholder expedited target selection in some form, which in turn facilitated the selection of the colour-defined half-circle. For example, the placeholder rendered the temporal onset of the target more predictable relative to the placeholder absent condition (the placeholder-to-target SOA was constant). This would reduce the trial-by-trial temporal variation of the initial GFBA modulation onset and result - as observed here - in a bigger response when averaging over trials. Less temporal jitter, however, would also predict a narrower temporal range of the modulation as described Luck^47^ (pp. 135–136), which is apparently not the case (cf. Fig. 5b). Still, the better temporal preparation could account for the decrease in reaction time and might have enhanced early visual stimulus processing (P1, N1)^57,58^. Such general processing enhancements, however, will equally effect matching and non-matching colors^57^ and should, therefore, not show up in the GFBA difference waveform. Another possibility is that constraining the spatial FOA to the placeholder area rendered colour-based selection of the target more effective. An account of this idea could be phrased in terms of the normalization model of visual attention^59^. One could assume that on target onset the spatial FOA is perfectly aligned with the target location in the placeholder condition (target and distractor colour in the focus), but not in the no placeholder condition, where attention is less focused, such that the FOA also contains (at least temporarily) the background colour in addition to the target and distractor colour. According to the normalization model, input from the attended area imposes a suppressive drive onto the response. Since three colours (target, distractor, and background) would generate a bigger suppressive drive than two colours (target and distractor), the brain response would be smaller in the placeholder absent condition, as observed here.

### Conflicting evidence from unattended probe paradigms

A couple of early EEG studies report no or only small feature selection effects for spatially unattended probes^4,11,12^. This is in direct contrast to the current experiments, which clearly show that prominent GFBA modulations appear under conditions of strong and consistent spatial focusing to the target and away from the probe. For discussing these conflicting observations, it seems to be important to consider other critical differences in the experimental designs. One difference is that most experiments reporting GFBA effects used longer stimulus durations than studies that failed to observe GFBA modulations. That is, those finding effects presented the target and probe for at least 300 ms, or they flashed 100 ms probes while the target was continuously presented e.g.,^7,8,17,27,29–31,33^. Hence, a minimum duration of stimulus presentation may be necessary to allow for an location-independent implementation of GFBA. Second, studies that did not find effects of GFBA, never presented the probe and target simultaneously on the screen. For example, Hillyard and Münte^11^ flashed a vertical colour bar for 32 ms either in the left or right visual hemifield (randomly). Subjects were to attend to one of the hemifields and discriminate the length of a bar drawn in the target colour. A bar with the target colour presented in the opposite unattended hemifield did not elicit a significant selection negativity (an index of feature selection). It is possible that in the absence of the target, i.e., without the process of selecting the colour-defined target, GFBA responses are small or inconsistent. In line with this reasoning, Bartsch, *et al*.^8^ found effects of GFBA to be diminished when the respective target colour was not present in the FOA (i.e., not part of the currently-attended object). Also, given that in those early EEG studies the target colour appeared either in the target or non-target visual field, the subjects may have used a strategic suppression of the items appearing in the unattended visual field to aid performance. This would naturally minimize or eliminate the probe’s potential to reveal GFBA effects. Specifically, such suppression could be associated with a P_D_ – a positive going modulation reflecting distractor suppression^60–64^ (see also discussion below) that would potentially reduce or cancel any selection negativity that may have been present for the unattended visual field.

### Global feature-based attention or attentional capture?

It is well-known that salient distractor items can attract attention, especially when they match the current task-set, such as an attended colour^65–67^. Given that the probe onset transient potentially disrupted previous spatial focusing^36,68^, attention could have been attracted to probes that match the attended feature immediately after stimulus onset. Although we did indeed observe slightly prolonged response times for probes matching the target colour (match trials 3–9 ms slower compared to non-match trials), this response time difference is much smaller than that expected by classical capture paradigms^65,66^ or attentional dwell–time literature^69^, which would both predict a response time prolongation of at least 50 ms. Furthermore, the response time difference between match and non-match trials is less pronounced for the conditions facilitating a stronger spatial focusing (“hard” condition of Experiment 1, “placeholder present” condition of Experiment 2). That is, if the observed modulation sequence would stem from occasional attentional capture events, it should be reduced (and not enhanced) for those conditions. As previously discussed in Bartsch *et al*.^8^, the slight response slowing on match trials is more likely explained by an issue of stimulus-response mapping (see Supplementary Data S1 for details). However, even though the behavioural data do not show substantial capture effects, subjects might have actively suppressed target colour probes to avoid being captured by them^70^. Specifically, recent EEG and MEG studies of attentional selection in visual search reported a component called distractor positivity or P_D_, which reflects such suppression of salient distractors^60–64^. It is theoretically possible, therefore, that the brain response to the probe could reflect such suppression mechanism instead of a global feature enhancement. However, the neuromagnetic fields reported here do not seem to be compatible with a P_D_^71^. Moreover, previously-reported EEG correlates showed a negative and not positive voltage deflection contralateral to the probe, see discussion in Bartsch, *et al*.^8^. Last but not least, attentional capture effects decrease with distance to the spatial FOA^72^, such that the probe that is presented quite distant from the target side (>5° between inner edges of target and probe) should not have attracted much attention. Taken together, it is unlikely that the reported modulation sequence is caused by spatial capture events. Of course, the present data cannot rule out that spatial selection influences GFBA in some form under other experimental conditions, where the target and probe location are less consistently defined.

## Conclusion

Our results suggest that GFBA, as measured with the unattended probe paradigm, does not arise as a consequence of inconsistent spatial focusing. Instead, motivating stronger pre-focusing on the target side enhanced initial parts of the GFBA modulation sequence. The spatial focus of attention should therefore be carefully controlled to maximize GFBA effects, and to best separate effects of attention to features from that of attention to space. The reported experiments confirm that feature-based attention operates in a spatially global manner across the whole visual field and can be measured with feature probes presented outside the current spatial FOA.

## Electronic supplementary material

Supplementary Information

## Data Availability

The datasets generated during and/or analysed during the current study are available from the corresponding author upon reasonable request.
