# Supplementary material for: Enhanced spatial focusing increases feature-based selection in unattended locations
Source: Sci Rep. 2018 Oct 31;8:16132. doi: 10.1038/s41598-018-34424-5 (PMC6208401; doi:10.1038/s41598-018-34424-5)
Supplement: Supplementary file 1 — Supplementary Information [file 41598_2018_34424_MOESM1_ESM.pdf]

Supplementary Information

(Article in Scientific Reports)

Enhanced spatial focusing increases feature-based selection in unattended locations

Mandy V. Bartsch[1,2,*], Sarah E. Donohue[1,2], Hendrik Strumpf[2], Mircea A. Schoenfeld[1,2,3], Jens-Max Hopf [1,2]

[1] Leibniz Institute for Neurobiology, 39118 Magdeburg, Germany
[2] Department of Neurology, Otto-von-Guericke University, 39120 Magdeburg, Germany
[3] Kliniken Schmieder Heidelberg, 69117 Heidelberg, Germany.

Supplementary Data S1: Behavioural performance data separately analyzed for trials with target half circles being presented on the left or right of the bicoloured circle (Exp. 1/2) as well as for being convex or concave (Exp. 2 only)

Both Experiment 1 and 2 showed prolonged response times when the probe matched the target colour (match trials). The slight response slowing for match compared to non-match trials was also found in previous experiments and might be most likely explained by an issue of stimulus-response mapping as discussed in the Supplementary of Bartsch, et al. [1]. In short, the experiments mapped left-right response alternatives to left-right targets, which might have interfered with the relative spatial position of a target colour probe presented on the right side of the screen prompting to a “right” response (given that color is processed throughout the whole visual field (GFBA)). Specifically, responses to target half circles that were on the left side of the circle (“target colour is on the left”) were slower and less accurate if the probe in the right visual field (VF) also contained the target colour (thereby providing some sort of incongruent “target colour is on the right” cue). Consequently, a probe in the right VF matching the target colour could also lead to faster and more accurate responses when it was consistent with the relative position of the target in the bicoloured circle (“target colour is on the right”). When comparing match and non-match trials, the slower “left” responses for match trials were not fully compensated by speeded “right” responses leading to the overall slowing on match compared to non-match trials. The data of the current experiments reveal a similar influence of the stimulus-response mapping on the performance data as discussed below.

Experiment 2, that uses the same response mapping structure than the experiments of Bartsch, et al. [1] (“left” targets require “left” response, “right” targets require “right” response), perfectly resembles the previously observed pattern with “left” responses being slower and less accurate on match trials compared to non-match trials, and “right” responses being faster and more accurate (see Supplementary Figure S1). The effect was apparent in both placeholder absent and placeholder present trials. Three-way rANOVAs with the factors

PLACEHOLDER (present/absent), MATCH (match/non-match) and SIDE (target on the left/right) confirmed that there was a significant SIDExMATCH interaction for both response time and response accuracy, but no three-way interaction of SIDExMATCHxPLACEHOLDER (Statistical parameters are summarized in Supplementary Table S1). Post-hoc pairwise comparisons (t-tests) between match and non-match trials confirm slower and less accurate responses for "left" targets as well as faster and more accurate responses for "right" targets (all p's ≤ 0.028). On match trials, slower "left" responses are not fully compensated by faster "right" responses leading to the slowing on match trials visible in Figure 2 of the main text.

Experiment 1, adopted a more complex response assignment. Specifically, "left" targets did not require a simple "left" response, but the subject had to report its curvature (convex: index finger, concave: middle finger). Although in the easy task, responses to targets on the left side were still slower for match compared to non-match trials, other spatial response mapping effects are even more prominent. Specifically, independent of the probe match, subjects responded slowest and least accurately to "left" targets that are concave. This effect is apparent on both easy and hard trials and is probably caused by an incompatibility of the spatial relation between target and response akin to the "Simon effect" [2-4]. That is, the position of the target in the left visual field, as well as its "left" position in the bi-coloured circle both prompt the subject to use the response alternative located on the left of their hand (index finger). Having then to respond with the middle finger (concave item) positioned to the right of the index finger led to particularly slow and error-prone responses. Four-way rANOVAs with the factors DIFFICULTY (easy/hard), MATCH (match/non-match), SIDE (target on the left/right), and CURVATURE (convex/concave) confirm a significant interaction of SIDExCURVATURE for both response time and response accuracy that was not influenced by MATCH (no significant three-way interaction). The statistical parameters are summarized in Supplementary Table S1. For response accuracy only, there was a significant three-way interaction of SIDExCURVATURExDIFFICULTY, which might reflect the drop in accuracy for concave "left" targets being even more pronounced on hard trials. Post-hoc pairwise comparisons confirm that responses are slowest and least accurate to concave "left" targets for hard match and hard and easy non-match trials (all p's ≤ 0.028, except for easy non-match "left" concave being not significantly less accurate compared to "right" convex: $p = 0.85$). For easy match trials some of the comparisons fail to reach significance with "left" concave not being significantly faster than "left" convex or "right" convex (both p's ≥ 0.128) and being not significantly different in terms of accuracy from "right" convex ($p = 0.26$). All other comparisons for easy match trials are significant (all p's ≤ 0.04).

Taken together, the pattern of the behavioural data imply that the slowing on match trials does not reflect an attentional selection of the probe (which should lead to a general impairment on match trials), but rather arises at higher levels of stimulus-response mapping and execution.

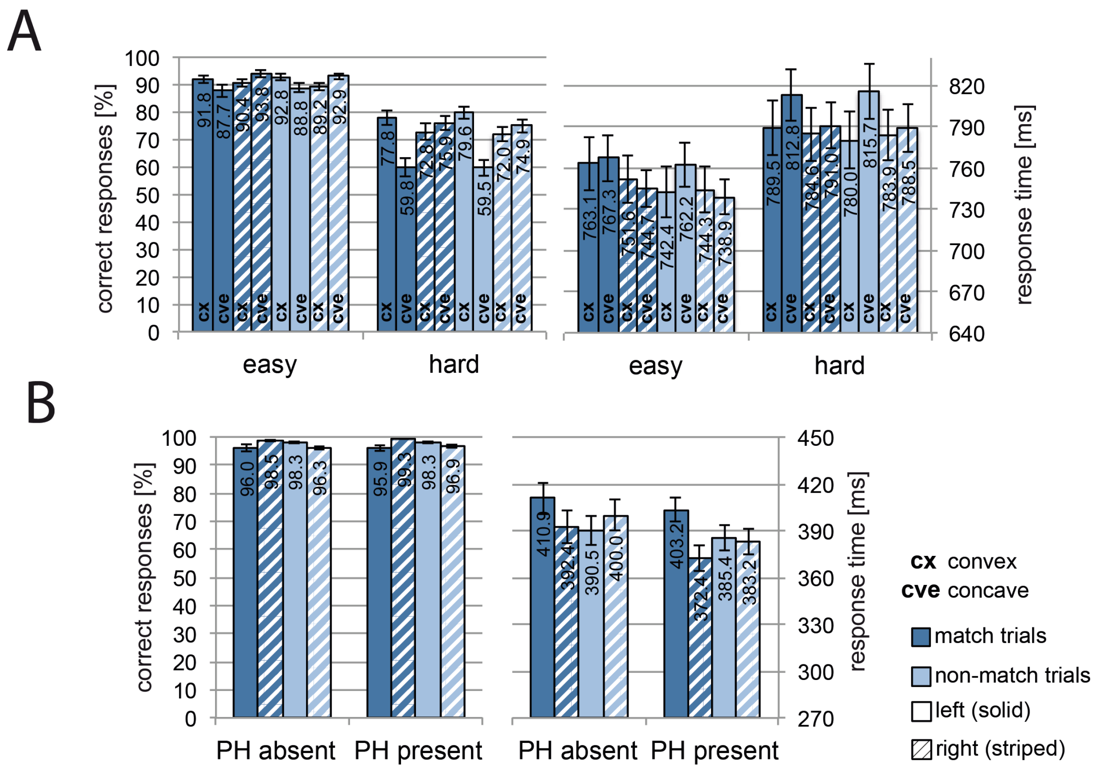

Supplementary Figure S1. A) Behavioural data of Experiment 1 for easy and hard trials split up in the target half-circle being on the "left" or "right" of the bicoloured cirle as well as it being "convex" (index finger response) or "concave" (middle finger response). B) Behavioural data of Experiment 2 for placeholder (PH) present and absent trials split up in target half-circles being on the "left" (index finger response) or "right" (middle finger response) of the bicoloured cirle. The error bars show the standard error of the mean. As can be seen, on match trials, responses for "left" targets are slower and less accurate than that for "right" targets. For Experiment 1, there is an additional response conflict most prominent for "left" targets that are concave and thus have to be answered with a button press of the middle finger (which is positioned "right" to the index finger).

Supplementary Table S1. (* p≤0.05, ** p≤0.01, *** p≤0.001, n.s. = not significant). Displayed are the results of the rANOVAs performed on the behavioural data.

		Response time			Response accuracy		
		**df**	**F**	**p**	**df**	**F**	**p**
**Exp. 2**	**PLACEHOLDER**	1,18	13.8	**	1,18	2.6	n.s.
	**MATCH**	1,18	27.1	***	1,18	0.004	n.s.
	**SIDE**	1,18	12.9	**	1,18	1.7	n.s.
	**PLACEHOLDER xMATCH**	1,18	5.0	*	1,18	0.02	n.s.
	**PLACEHOLDER xSIDE**	1,18	23.2	***	1,18	5.8	*
	**MATCHxSIDE**	1,18	152.5	***	1,18	14.1	**
	**PLACEHOLDER xMATCHxSIDE**	1,18	0.09	n.s.	1,18	0.04	n.s.
**Exp. 1**	**DIFFICULTY**	1,18	12.7	**	1,18	165.3	***
	**MATCH**	1,18	22.3	***	1,18	0.008	n.s.
	**SIDE**	1,18	9.8	**	1,18	5.9	*
	**CURVATURE**	1,18	3.8	n.s.	1,18	5.0	*
	**DIFFICULTYxMATCH**	1,18	10.3	**	1,18	0.034	n.s.
	**DIFFICULTYxSIDE**	1,18	0.07	n.s.	1,18	6.3	*
	**MATCHxSIDE**	1,18	1.8	n.s.	1,18	13.2	**
	**DIFFICULTYxMATCHxSIDE**	1,18	0.8	n.s.	1,18	0.11	n.s.
	**DIFFICULTYxCURVATURE**	1,18	12.7	**	1,18	17.0	***
	**MATCHxCURVATURE**	1,18	7.5	*	1,18	0.35	n.s.
	**DIFFICULTYxMATCHxCURVATURE**	1,18	0.47	n.s.	1,18	0.56	n.s.
	**SIDExCURVATURE**	1,18	12.4	**	1,18	23.1	***
	**DIFFICULTYxSIDExCURVATURE**	1,18	0.48	n.s.	1,18	6.4	*
	**MATCHxSIDExCURVATURE**	1,18	2.8	n.s.	1,18	0.35	n.s.
	**DIFFICULTY xMATCH xSIDExCURVATURE**	1,18	0.0001	n.s.	1,18	0.26	n.s.

Supplementary Data S2: Statistical analyses of probe responses outside the GFBA time windows

The statistical analyses of probe responses focused exclusively on time ranges of significant global feature-based attention (GFBA) effects (i.e., significant differences between match and non-match trials). However, there might be condition-specific differences (Exp. 1: easy versus hard; Exp. 2: placeholder absent vs. present) outside those time windows that influence the observed global feature effects. In particular, the physical presence of a placeholder in Exp. 2 will elicit an additional ERP response. Such factors should be the same among match and non-match trials and, hence, be eliminated in the respective difference waveform indexing GFBA. However, we cannot exclude the possibility that the ERP response evoked by the placeholder interacts later on with the probe match before or after the reported GFBA modulation. To investigate this issue, we ran additional analyses to search for interactions between probe match and task difficulty (Exp. 1) as well as probe match and placeholder presence (Exp. 2) outside the pre-defined early and late GFBA time windows.

Experiment 1: 30ms-sliding-window rANOVAs with the main factors DIFFICULTY (easy/hard) and MATCH (match/non-match) were performed from 0ms to 500ms after target onset on sensors of the early and late GFBA response. There was, indeed, a main effect of DIFFICULTY on the sensors displaying the late probe response around 451-477ms ($p < 0.05$, corrected for multiple comparisons, as described in Methods). It did, however, not interact with the probe matching condition.

Experiment 2: Since the baseline was not comparable between placeholder present and absent trials (the placeholder presence evoked an additional ERP that overlapped with the baseline of following stimuli), we could not fairly analyse these data with a full rANOVA design, and therefore evaluate the influence of placeholder presence on the match minus non-match (M-NM) difference waveforms (that subtraction gets rid of the baseline issue since it is the same for match and non-match trials) with a 30ms-sliding-window t-test between 0ms to 500ms after target onset. If the placeholder presence interacts with effects of GFBA, the M-NM waveforms indexing GFBA should significantly differ between placeholder present and absent trials. The only significant difference between placeholder present and absent M-NM waveforms was found on the sensors displaying the early GFBA effect within the time range of the early GFBA modulation (233-241ms) ($p < 0.05$, corrected for multiple comparisons, as described in Methods).

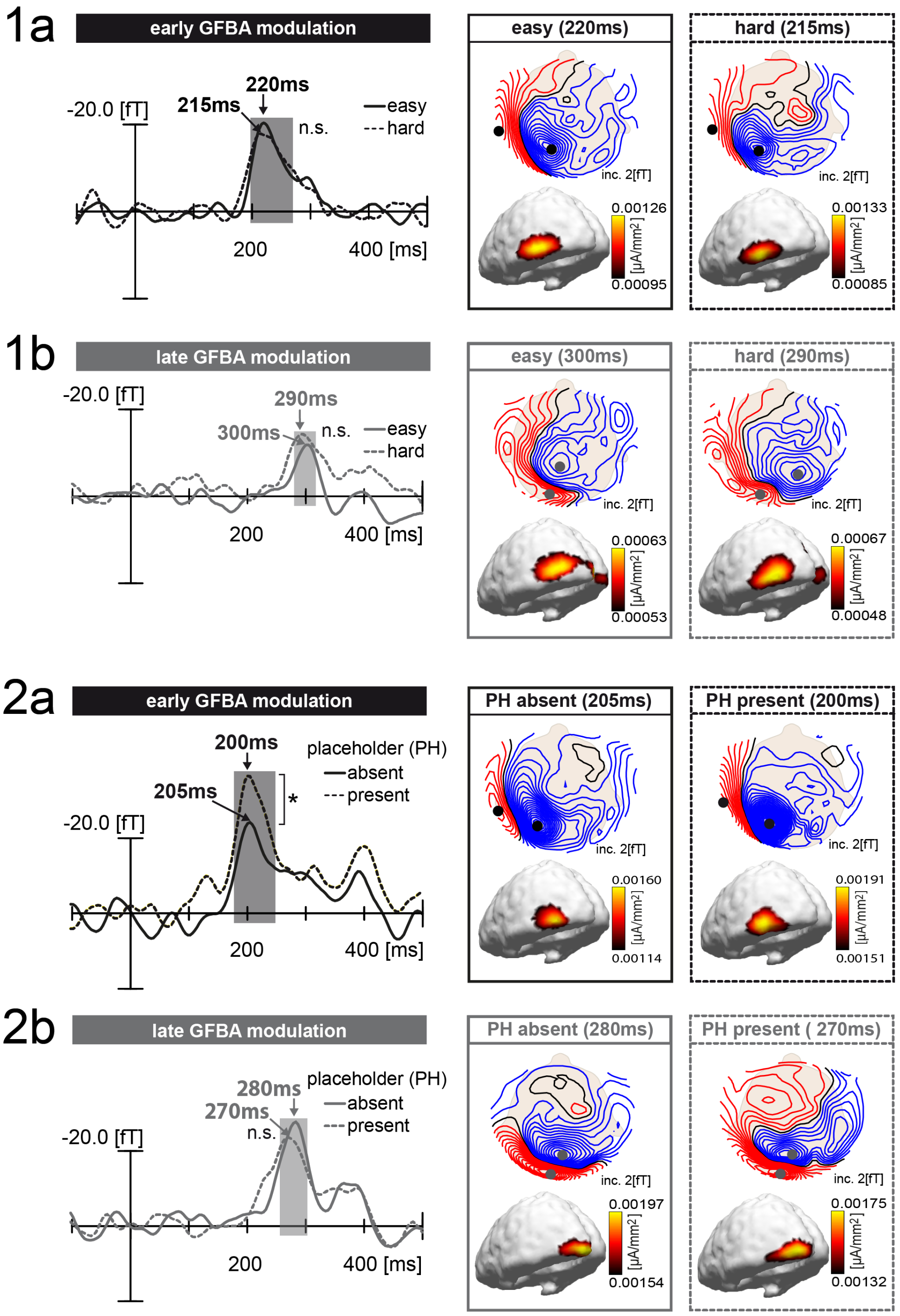

Supplementary Figure S2. Shown are the early and late GFBA effects (M-NM difference) for Experiment 1 (1a, 1b) and Experiment 2 (2a, 2b) with sensor sites chosen at field effect maxima of individual conditions (i.e., sensors sites can now differ between easy/hard (Exp. 1) or placeholder present/absent (Exp.2) conditions). As can be seen, for Exp. 2, the sensor choice did only marginally change (early waveform of placeholder absent (black solid in 2a) slightly different to that of Figure 5b in the main manuscript). For Exp. 1, sensors sites are now apparently better adjusted to the individual field maxima of the late modulation (compare 1b with Figure 3c in the main manuscript). However, none of the effects reported in the manuscript changed qualitatively. In the time ranges of GFBA modulation, easy and hard M-NM waveforms do still not differ (both $p \geq 0.38$), placeholder absent and present M-NM waveforms differ in the early ($p = 0.0023$), but not late time window ($p = 0.47$) as reveald by pairwise comparisons (t-tests).

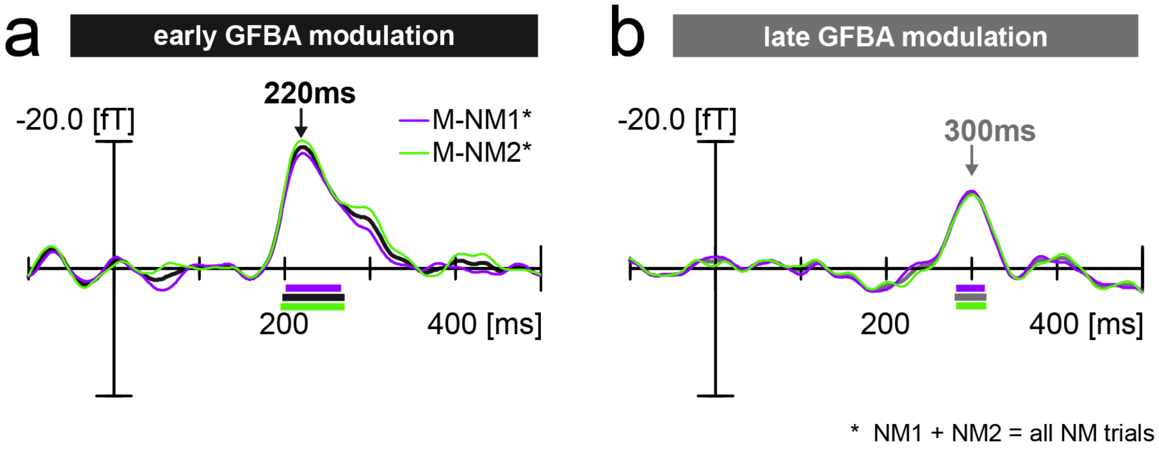

Supplementary Figure S3. Comparing match (M) and non-match (NM) trials of equal trial numbers. Shown are the ERMF waveforms of the early (a) and late (b) GFBA modulations of Experiment 1 (averaged across easy/hard conditions). Since there were twice as many non-match trials compared to match trials, non-match trials were splitted into two subsamples (NM1 and NM2) of equal trial numbers. Specifically, non-match sub-bins (e.g., target color is red, probe color blue) were randomly distributed between NM1 and NM2 with this restriction that target and probe colors were balanced between NM1 and NM2 (i.e., same amount of red probes in both bins, etc.,). The respective M-NM1 (purple line) and M-NM2 (green line) waveforms are plotted together with the M-NM data from Figure 3a in the main text (black and grey lines). Horizontal bars indicate the respective time windows of significant match vs. non-match comparisons ($p < 0.05$, corrected for multiple comparisons, as described in Methods). As can be seen, the M-NM difference waveform is preserved when taking half the amount of non-match trials. Hence, the GFBA modulation sequence (M-NM) does not arise as a consequence of an imbalance in trial number between match and non-match trials.

References

1 Bartsch, M. V. *et al*. Determinants of Global Color-Based Selection in Human Visual Cortex. *Cereb. Cortex* **25**, 2828-2841, doi:10.1093/cercor/bhu078 (2015).

2 Nicoletti, R., Anzola, G. P., Luppino, G., Rizzolatti, G. & Umiltà, C. Spatial compatibility effects on the same side of the body midline. *J. Exp. Psychol. Hum. Percept. Perform.* **8**, 664-673 (1982).

3 Nicoletti, R. & Umiltà, C. Attention shifts produce spatial stimulus codes. *Psychol. Res.* **56**, 144-150 (1994).

4 Nicoletti, R., Umiltà, C. & Ladavas, E. Compatibility due to the coding of the relative position of the effectors. *Acta Psychol. (Amst.)* **57**, 133-143 (1984).
